# Genome diversity and instability in human germ cells and preimplantation embryos

**DOI:** 10.1016/j.semcdb.2020.12.007

**Published:** 2021-05

**Authors:** Vallari Shukla, Miya Kudo Høffding, Eva R. Hoffmann

**Affiliations:** DNRF Center for Chromosome Stability, Department of Cellular and Molecular Medicine, Faculty of Health and Medical Sciences, University of Copenhagen, Denmark

**Keywords:** Genome instability, Aneuploidy, CNVs, Rearrangements, Human oocytes and embryos, Genomic disorders, DNA damage response

## Abstract

Genome diversity is essential for evolution and is of fundamental importance to human health. Generating genome diversity requires phases of DNA damage and repair that can cause genome instability. Humans have a high incidence of *de novo* congenital disorders compared to other organisms. Recent access to eggs, sperm and preimplantation embryos is revealing unprecedented rates of genome instability that may result in infertility and *de novo* mutations that cause genomic imbalance in at least 70% of conceptions. The error type and incidence of *de novo* mutations differ during developmental stages and are influenced by differences in male and female meiosis. In females, DNA repair is a critical factor that determines fertility and reproductive lifespan. In males, aberrant meiotic recombination causes infertility, embryonic failure and pregnancy loss. Evidence suggest germ cells are remarkably diverse in the type of genome instability that they display and the DNA damage responses they deploy. Additionally, the initial embryonic cell cycles are characterized by a high degree of genome instability that cause congenital disorders and may limit the use of CRISPR-Cas9 for heritable genome editing.

## 1 Introduction

Genome diversity is generated from DNA damage and repair processes in response to endogenous and exogenous causes. The DNA damage response (DDR) is highly conserved and consists of at least nine different repair pathways and over 700 genes, several with overlapping functions [1]. DNA damage triggers a range of cellular responses including cell cycle arrest and repair of the lesion, as well as senescence, autophagy and apoptosis. The repair event can lead to genomic changes, from single nucleotide variants to structural and copy number variants or more substantive whole chromosome gains and losses (aneuploidy).

Until recently, most of our knowledge on genome diversity and instability in humans has been from population studies. In 1959, aneuploidies were first identified in individuals with developmental syndromes and congenital disorders such as those associated with Klinefelter, Turner and Down syndromes [2], [3], [4], [5], [6]. Subsequent studies in fetal losses revealed that human conceptions have exceptionally high levels of aneuploidy, particularly in association with maternal age [7]. As a result, countries across the world have implemented prenatal screening programs [8].

In the past decade, the rapid development in sequencing technologies [9] together with biobank and cohort consortia have facilitated large sequencing studies. This has led to current estimates that up to 8% of children are born with a predisposition to genetic conditions that may result in serious diseases or disorders (Table 1). Over 4000 genes with rare single nucleotide variants and around 70,000 quantitative trait loci have been identified that are associated with specific disease phenotypes in humans [10]. Increasingly, *de novo* structural variants including insertions/deletions and copy number variants (> 50nt) have been shown to be important in genetic disorders [11], [12], [13], [14]. Moreover, the incidence of *de novo* structural variants are orders of magnitude higher than *de novo* single nucleotide variants in the general population [15]. Rare cases of severe genome rearrangements including chromothrypsis and multiple *de novo* copy number variants have also been identified in pediatric patients with developmental disorders. They appear to arise independently of single copy number variants [16]. Of ClinVar entries, 10% of SNVs are listed as pathogenic or likely pathogenic, whereas for copy number variants this increases to 41%; however, the majority of entries are variants of unknown significance. Successful population screening programs have largely been confined to Centers for Disease Control Tier 1 genetic disorders, such as autosomal dominant monogenic disorders with known pathologies, clinical actionability, and implications for population health [17].

**Table 1:** Genomic alterations and associated disorders in live births.

Genomic alteration^a^	Description	Example of genomic disorders^b^	Incidence^c^
Aneuploidy	Whole chromosomal aneuploidies	Down syndrome, Triple X syndrome, Klinefelter syndrome, Turner syndrome, Edwards syndrome, Patau syndrome	1 in 1000–1 in 10,000
CNVs	Chromosomal deletions, insertions, duplications translocations and rearrangements	Whole arm deletions: Jacobsen syndrome, 6q deletion syndrome, Cri-du chat syndrome	1 in 3000–1 in million
		Deletion of imprinting genes: Prader-Willi syndrome, Angelman syndrome	
		Duplications and deletions due to NAHR: Charcot-Marie-Tooth disease type 1A, Gaucher Disease, Hunter syndrome	
		INDELs: Cystic fibrosis, Fragile X syndrome, Parkinsons disease	
SVs	Structural variants smaller than 50 bp	Psoriasis, autism and schizophrenia	Rare
SNVs	Single nucleotide polymorphisms	Mutations in various DNA damage response and repair genes: Li-fraumeni syndrome, Progeria, Fanconi anemia, Lynch syndrome, Xeroderma pigmentosa, Familial breast cancer, Ataxia telangiectasia	1 in 5000 to less than 1 in 1 million
Rare *de novo* mutations	Sporadic genetic mutations	Schinzel–Giedion syndrome, Kabuki syndrome, Bohring–Opitz syndrome, Proteus syndrome	Less than 1 in 1 million
Complex chromosomal rearrangements	Multiple chromosomal aberrations and rearrangements	Isodicentric Y chromosome formation, ring chromosomes	Very rare
Balanced chromosome rearrangements	Reciprocal translocations between two different chromosomes	Neurodevelopmental and psychiatric disorders; developmental disorders	1 in 600

Abbreviations: INDELs-insertions-deletions; NAHR-non-allelic homologous recombination; CNVs-copy number variants; SNVs-single nucleotide Variants; SV-structural variants.

a *de novo* alterations.

b Indels are < 1 Kbp. Large CNVs examples are larger than 500 Kbp. Examples of genomic disorders are those from live birth.

c *de novo* incidence amongst live births from the NIH National Human Genome database.

Genome instability leading to *de novo* single nucleotide variants, insertion/deletions, *de novo* copy number variants and aneuploidies are inferred to occur both in the germline [13] as well as during the mitotic divisions in the preimplantation embryo [16], [18], [19]; however, the vast majority of these genomic constellations are unlikely to be seen in live births. 25% of all clinically-recognized pregnancies end in loss and at least half of these are due to aneuploidy, including those affecting chromosomes that are not seen in live births [20]. Continuous improvements of single-cell sequencing technologies are providing new insights into the origins and incidence of genomic changes that are generated in human germ cells and preimplantation embryos [21], [22] (Fig. 4; Table 2). With increased genetic testing of preimplantation embryos, new estimates suggest that at least half of blastocyst embryos have genomic alterations that may explain the high incidence of preclinical pregnancy losses [23]. These are mainly aneuploidies as well as very large copy number variants (10 Mpb or larger). More recently, CRISPR-Cas9 has been used for genome editing in human embryos; whilst its use for heritable genome editing remains highly controversial, as a research tool it will facilitate our understanding of DNA repair capacity in human embryos [24], [25], [26].

**Fig. 1:**
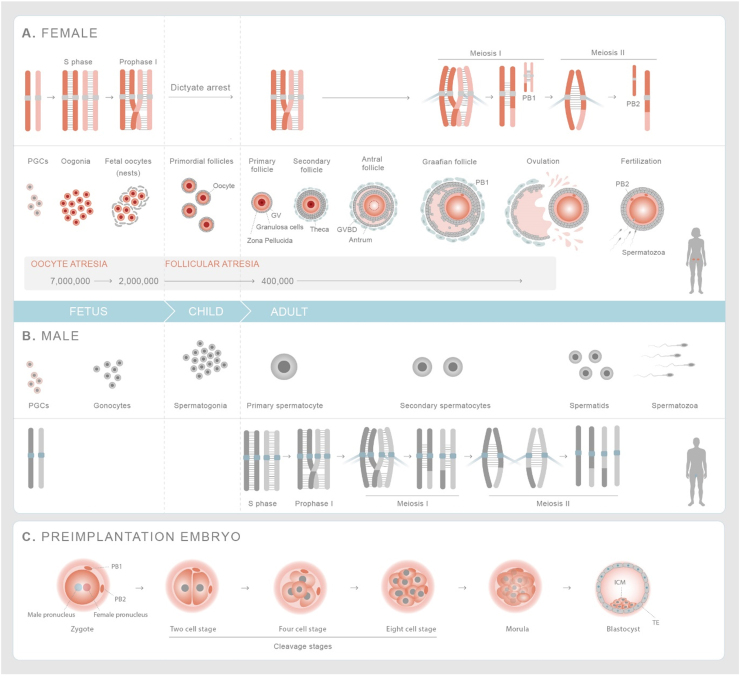
Germline and preimplantation embryo development in humans. (A) Female-Primordial germ cells expand and are specified as oogonia, which form nests before initiating meiosis. Fetal oocytes replicate their DNA and homologous chromosomes undergo meiotic recombination before entering dictyate (G2/M) arrest. Prior to birth, individual oocytes are surrounded by layer of follicular cells forming primordial follicles. Follicles are recruited throughout life but only after onset of puberty do they mature over 290 days. Once per month, the luteinizing hormone surge causes the ovulation of a single mature egg that has resumed and completed the first meiosis division. At this stage, the cohesion between sister chromatid arms are released and homologous chromosomes segregate forming the mature, secondary oocyte and first polar body (PB1). Upon fertilization the sister chromatids separate giving rise to zygote and second polar body (PB2). (B) Male-The primordial germ cells are specified as gonocytes at fetal stage, which differentiate into spermatogonia upon birth. Meiosis is initiated at the onset of puberty in primary spermatocytes and completed in round spermatids. Round spermatids undergo morphological differentiation to give rise to haploid sperm. (C) Preimplantation embryos-Zygote consists of female and male pronuclei that fuse together and undergo first mitotic division. After initial 2–3 divisions, the cells undergo compaction forming a morula. Subsequent divisions lead to the formation of blastocyst which comprises of a cavity (blastocoel) and inner cell mass with a layer of trophectoderm cells around it. PB1 and PB2 chromosomes are shown in smaller size to indicate that their extrusion after MI and MII division respectively stops their transmission into germline. List of abbreviations - PGCs-Primordial germ cells; GV-Germinal Vesicle; GVBD-Germinal Vesicle Breakdown; TE-Trophectoderm; ICM-Inner Cell Mass.

**Fig. 2:**
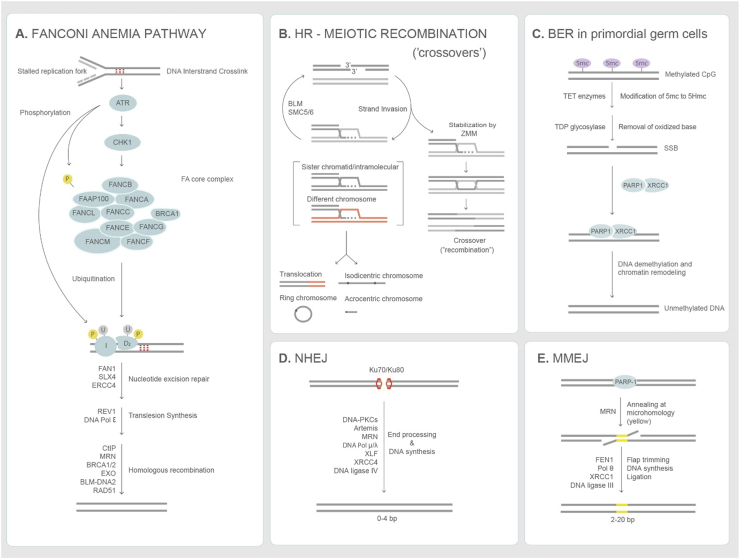
DNA damage repair pathways. (A) Fanconi Anemia pathway described from various studies in somatic cells and *Xenopus* extracts. Stalled replication forks activate ATR mediated checkpoint response, which recruits the FA core complex, which monoubiquitinates the FANCI/FANCD2 complex. The FANCI/D2 complex with SLX4 promotes the nucleolytic initiation, interstand unhooking and double strand break formation. DNA replication is resumed by translesion synthesis polymerases. The double strand break end initiates resection and repair via homologous recombination [49]. (B) Homologous recombination-meiotic recombination is initiated by SPO11-induced double strand breaks. Invasion structures are based on findings in budding yeast (see text). ZMM-complex of meiosis-specific proteins that are conserved and promotes double Holliday junction formation and their biased resolution into crossovers [203], [204]. Invasion structures are unwound/disassembled by BLM and SMC5/6 and can re-populate the meiotic recombination pathway (crossover or synthesis-dependent single strand annealing for noncrossovers-not shown). Invasion structures that are not disassembled can be resolved by structure-specific endonucleases, yielding both crossovers and noncrossovers [205]. (C) The Base excision pathway is required by murine primordial germ cells [40] and the pronucleus of embryos [196] to undergo demethylation, which is essential for gametogenesis. TET enzymes oxidize 5mC to 5HmC, which is oxidized. TDP glycosylase removes the oxidized base creating a single strand break recognized by XRCC1 and PARP1 that facilitate DNA demethylation and chromatin remodeling. (D) Non-homologous end joining pathway (NHEJ) - Double strand break ends are recognized and processed by Ku70/80 heterodimer, DNA-PKCs and other enzymes. The ends are ligated by DNA ligase IV/XRCC4/XLF [206]. (E) Microhomology mediated end joining pathway (MMEJ) uses short homologous sequences (shown in yellow) to align the broken ends. FEN1 mediates flap trimming and the ends are ligated by XRCC1/DNA Ligase III [207]. End joining pathways are preferred double strand repair mechanisms in preimplantation embryos [24], [25], [26]. List of abbreviations - BER-base excision repair; NHEJ-nonhomologous end joining; MMEJ-microhomology mediated end joining; HR-homologous recombination. (For interpretation of the references to color in this figure legend, the reader is referred to the web version of this article.)

**Fig. 3:**
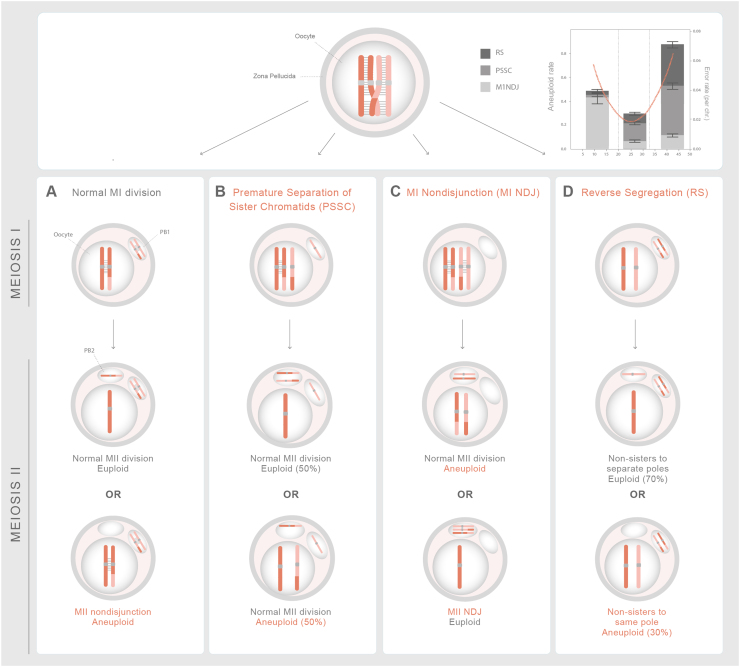
Segregation error patterns in human oocytes follows a U-curve with female age. (A) Normal MI division the homologous chromosomes segregate forming the MII oocyte and the first polar body. Normal MII division, where sister chromatids disjoin is shown below as is a MII nondisjunction event leading to an aneuploid conception. (B) During premature separation of sister chromatid (PSSC) one sister chromatid separates from its homolog and ends up either in oocyte or PB1 leading to aneuploidy after MI division. The segregation of the single chromatid in meiosis II results either in a euploid or an aneuploid conception. Meiosis II errors occur independently and would result in an aneuploid conception. (C) Non-disjunction (MI NDJ) of homologous chromosmes during MI division leads to aneuploidy with either the oocyte or the PB1 ending up with both chromosomes. A normal meiosis II division results in an aneuploid conception, whereas a meiosis II error can restore, by chance, the euploid maternal genome. (D) Reverse segregation where sister chromatids of both homologous chromosmes separate during MI division. The segregation of the two nonsister chromatids results in 70% euploid eggs after MII division [69], [91]. List of abbreviations – MI - Meiosis I division; MII - Meiosis II division; PB1- Polar Body 1; PB2 - Polar Body 2.

**Fig. 4:**
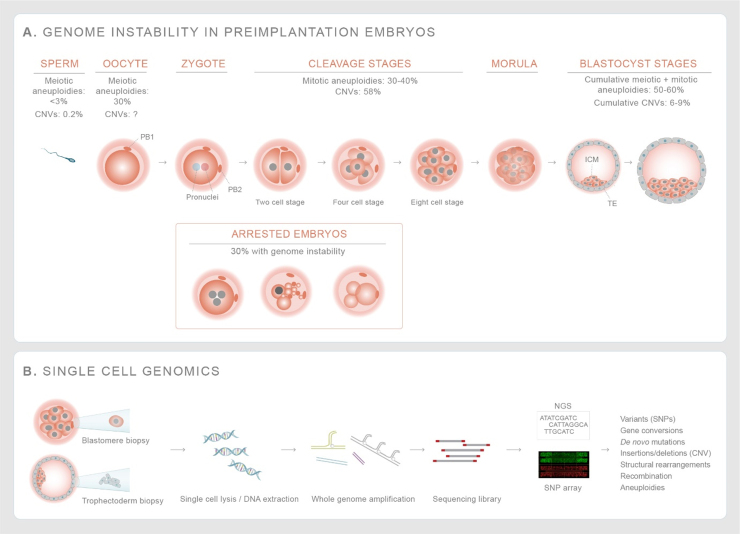
Phases of high genome instability during human embryogenesis – (A) The incidence of aneuploidy is higher in eggs (30%) than in sperm (< 3%). The incidence of mitotic aneuploidies and gross chromosomal rearrangements increase during cleavage and blastocyst stages. About 30% of human cleavage stage embryos arrest, with evidence of genome instability such as gross chromosomal rearrangements and complex aneuploidies that affect multiple chromosomes [138], [208], [209], [210], [211], [212], [213]. The cumulative aneuploidies from germ cells and mitotic divisions result in a high incidence of blastocyst embryos that are genomically mutated. (B) Use of single cell genomics technologies including whole genome amplification and sequencing of biopsied of cell(s) from cleavage stage embryos or blastocysts helps to map out gross chromosomal rearrangements and aneuploidies giving a better overview of genome instability in human preimplantation embryos. List of abbreviations- CNV - Copy number variants; NGS - next generation sequencing; SNP - single nucleotide polymorphisms.

**Table 2:** Incidence of genomic alterations at various developmental stages.

Genomic alteration	Eggs^a^	Sperm^b^	Preimplantation embryos-cleavage^c^	Preimplantation embryos- blastocysts^d^	Pregnancy loss^e^	Stillbirths^f^	Live births^g^
MLH1 foci	50 ± 24.7 (range: 10–107)	50 ± 4.1	–	–	–	–	–
Meiotic recombination rates (crossover rates)^a^	Ave: 76 (range: 27–124) (’trios’)	22–28	NA	Mat: 20–68	NA	NA	Mat: 38
							Pat: 24 ± 2.7
				Pat average: 24			
Whole chromosomal aneuploidy	30% (20–85% pending age)	2.5% (2.5–7%)	Up to 73%	56%^d^	50–60%	6.9%	1:1000
Large CNVs	7%	0.4%	58%	5.3%	NA	N.A	rare
(> 10 Mbp)							
SNVs	NA	2–4 × 10^-8^	NA	NA	NA	NA	Up to 70 pending paternal age; up to 10 pending maternal age [154]

Abbreviations: CNVs-copy number variants; SNVs-single nucleotide variants.

NA-not available.

a Recombination rates are provided as either genome wide (eggs and polar bodies ‘trios’ or for haploid genome). Refs. [77], [91]. Total eggs-PB: 77 from 13 donors.

b MLH1 foci in spermatocytes from Ref. [155]. Meiotic recombination rates from single haploid sperm sequencing from three studies and 22 donors. Total sperms: > 35,000 Refs. [116], [117], [118]. SNV rate from [117].

c Aneuploidies from Refs. [141], [144], [156]*.* Large CNV rates inferred from Refs. [140], [157].

d Paternal and maternal recombination rates inferred from 430 preimplantation embryos (blastocysts). Refs. [91], [98]. Large CNV and aneuploidy rates from over 8000 blastocyst embryos. Ref. [23].

e Pregnancy loss data from 1st trimester. Ref. [20].

f Aneuploidy in 532 Stillbirths [158].

g Live birth meiotic recombination rates computed from [159] in [117].

Here we review genome instability and the mechanisms that create genomic alterations in primordial germ cells to preimplantation embryos. We focus on identifying the endogenous causes of genome instability, the evidence that they are conserved in human germ cells and embryos, and their implications for reproductive phenotypes and human genetics.

## 2 Genetic diversity originates in the ‘immortal’ germline

The germline in sexually reproducing organisms is ‘immortal’ [27]. Only genetic changes that occur in the germline and their progenitor cells will be passed on to future generations. Primordial germ cells (PGCs) are the progenitors of egg and sperm (Fig. 1) and emerge around the second to fourth week of fetal development when gastrulation occurs in humans [28], [29]. Once specified, primordial germ cells migrate to the genital ridges until the 10th week where they then divide further as gonocytes or oogonia in the male or female gonad, respectively.

At six months after birth, gonocytes (also called as prospermatogonia) differentiate into spermatogonia, also known as spermatogonial stem cells. Humans have two types of spermatogonial stem cells; undifferentiated type A (A_dark_ and A_pale)_ and differentiated type B. Type A spermatogonia undergoes differentiation to form Type B spermatogonia, which undergo mitotic proliferation until early puberty. At puberty, spermatogonial stem cells differentiate into spermatocytes that enter meiosis and mature to produce haploid sperm in a process that lasts 74 days (Fig. 1-Male) [30], [31].

Primordial germ cells that enter the female fetal gonad expand and become oogonia, which initiate meiosis in asynchronous waves from the 12th to the 24th week of gestation [32]. Fetal oocytes initiate and complete (pre)meiotic S phase and meiotic recombination during prophase I prior to arresting at the dictyate stage (G2/M; Fig. 1-Female) [32]. Prior to birth, oocytes undergo nest breakdown and become surrounded by supporting cells (pre-granulosa cells) to form primordial follicles. This is associated with substantial germ cell loss. Of the 5–7 million oogonia, 2 million primordial follicles are left at birth and an estimated 250,000–500,000 are present at the onset of menarche (puberty), and depletion of the ovarian reserve occurs by 50–52 years (menopause). Throughout life, primordial follicles are recruited for growth; however, a single follicle matures to ovulate an egg once per month only from menarche until menopause (Fig. 1) [33]. At ovulation, the oocyte resumes and completes meiosis I, where homologous chromosomes segregate to reduce the genome content by half to form a haploid egg [34], [35]. Upon fertilization, the oocyte resumes meiosis II and segregates sister chromatids and the zygote is formed (Fig. 1-embryo). In human females, meiosis can last up to five decades and the extended dictyate arrest leads to a high degree of genome instability. This is in contrast to males, where stem cell renewal lasts a lifetime and is followed by a relatively short meiotic program to produce haploid sperm [34], [35].

A variety of fertility phenotypes in human are associated with the abrogation of the germ cells and preimplantation embryos (Table 3). Some of these phenotypes are similar to model organisms, allowing inferences to be made across species. Below, we discuss genome instability in the human germline, incorporating knowledge of both congenital conditions and bottom-up approaches from model systems where appropriate.

**Table 3:** Human fertility-related disorders and implicated factors.

Disorder or factor	Phenotype	Causes
Embryonic failure	Arrested embryos	Mutations in *WEE2*, *TUBB8*, *PLK4* genes [143], [160], [161], [162], [163]
Pregnancy loss, including recurrent pregnancy loss	Loss of pregnancy in first trimester (ICD-11 GA33)	> 50% are aneuploid; also large chromosomal rearrangements; triploid [20]
Testicular germ cell tumors	Tumors arising from pre-natal germ cells (gonocytes)	Isochromosome 12p [164]
Asthenozoospermia	Immotile spermatozoa in ejaculate	Primary ciliary dyskinesis, DNA fragmentation, elevated ROS levels [165]. Reviewed in [127]
Globozoospermia	Round headed, acrosome less spermatozoa, DNA fragmentation	Deletion of *DPY19L2* gene due to NAHR [166]
Macrozoospermia	Large headed and multiflagellated spermatozoa	Mutations in *AURKC* gene [167]
Non-obstructive azoospermia	Absence of spermatozoa in ejaculate. Chromosomal abnormalities, meiotic arrest	Mutations and deletions in *AZF* genes, *SYCP3*, *DMC1*, *SYCE1*, *MCM8*, *XRCC2*[127], [128], [168], [169], [170], [171], [172]
Sertoli cells only syndrome	Absence of germ cells	Mutations and deletions in *AZF* genes and *FANCA*[127], CNVs in *HOXD9, SYCE1, H19, COL1A1, KCNQ1*[173]
Oligozoospermia	Low sperm count, DNA fragmentation	Mutations and deletions in *AZF* genes, autosomal translocations and inversions [127], [168]
Advanced maternal age	Aneuploidy of eggs	Meiotic recombination GWAS signals (Down Syndrome); AurkB; cohesion loss [69], [102], [174], [175]
Oocyte maturation failure	Oocytes fail to mature to MII	Mutations in *WEE2*, *TUBB8*, cell cycle arrest at the GV stage, MI arrest due to meiotic spindle instability [160], [161], [162], [163]
Premature/primary ovarian insufficiency (POI)	Hypergonadotrophic hypogonadism; elevated *FSH* and low AMH levels, amenorrhea for at least four months before the age of 40 years (ICD-11 GA30.6)	Monogenic, high penetrance mutations is DNA damage response and repair genes including *BRCA1*, *BRCA2*, *CSB/ERCC6, FANCA, FANCM*, *FANCG, FANCL*, *MCM8*, *MCM9*, *HFM1*, *MSH4, MSH5, MEIOB, STAG3, SMC1β, SYCP2L, SYCE1, TP63.* Over 118 genes. Screening currently for pre-*FMR1* mutation [58]

Abbreviations: NAHR-non-allelic homologous recombination; CNV-copy number variants; MII-Metaphase II; FSH-follicle stimulating hormone; AMH-anti-Muller hormone.

## 3 Genome stability in primordial germ cells and other proliferative germ cells

Mouse models show that global epigenetic reprogramming and erasure of CpG methylation involves specific DNA repair pathways in primordial germ cells. DNA methylation sequencing of human primordial germ cells and other proliferative germ cells has revealed that this phase of development may contribute to C > T mutations at CpG sites.

### 3.1 Global epigenetic reprogramming requires base excision repair

Our understanding of genome instability in primordial germ cells in vivo in humans is incomplete due to the need for studies in early, developing human embryos and fetuses that are difficult to collect. However, recent developments of in vitro generation of human primordial germ cell-like cells (PGCLCs) may overcome this significant limitation [36], [37] and provide exciting new opportunities to understand genome stability in the earliest progenitors of gametes that we know of.

As primordial germ cells migrate and colonize the genital ridges around 3–4 weeks in fetal life, they erase their epigenome. This includes global DNA demethylation at CpG sites, genomic imprinting erasure and re-activation of their X chromosome (reviewed in [38]). In both female and male mice, primordial germ cells also initiate germline programming-responsive genes that are important for entry and completion of meiosis [39]. The hypomethylated state is characterized by chromatin-bound Xrcc1 (X-ray repair cross complementing 1) as well as poly(ADP)-ribose (PAR) polymerase, mechanistically linking base excision repair to extensive chromatin remodeling [40] (Fig. 2). The substrates for base excision repair are generated during global demethylation by the Ten-Eleven Translocation (TET) enzymes, which oxidize 5-methyl-cytosine to 5-hydroxymethyl-cytosine that can be further oxidized to 5-carboxyl-cytosine and 5-formylcytosine. The latter are substrates for thymine DNA glycosylase, which excises the oxidized base and generates an abasic site that is repaired by base excision repair or single strand break repair [40], [41], [42], [43] (Table 4) (Fig. 2-BER). Conditional knockout mice lacking *Xrcc1* in primordial germ cells are infertile and show depletion of primordial germ cells as well as loss of pluripotency markers [44]. Whether the regulation by base excision repair in reprogramming and the return to a naïve state in primordial germ cells are conserved in humans is unclear, but the developments of primordial germ cell-like cells should enable such questions to be answered.

**Table 4:** DNA damage response in mammalian germline and preimplantation embryos.

Cell/stage	DNA damage response/checkpoint^a^	Human condition^b^
Primordial germ cells	Base excision repair and single-strand break repair. Thymine DNA deglycosylase (TDG) generates abasic sites for BER, after TET-dependent oxidation of 5mC to 5hmC and 5fC and 5caC during genome-wide demethylation [40], [41], [42].	*PARP2* SNP implicated in ANM [58]
	ICL repair. Fanconi Anemia (*Fanca*) together with Xpf-Ercc1 and Slx4 may remove interstrand crosslinks generated by reactive aldehydes [48] and/or lipid peroxidation [176].	FA is associated with hypogonadism and POI [54], [53]
		XPF with POI [177]
	*Atm-p21-p53* dependent checkpoint during compromised replication in *Fancm*[46].	*ATM* is associated with POI [178]
	*Mcm9* mutants show primordial germ cell loss due to reduced proliferation, independently of Atm-Chek2-p21-p53 [179].	MCM9 variants associated with POI [180], [181]
	Extra spindle pole bodies 1 (separase) plays a critical role in maintenance of sister chromatid cohesion and genome stability in PGCs [182].	
	*Mad2l2* is required for PGCs development during epigentic reprogramming [183].	
	*Atrx/Rad54* knockout mice lead to loss of premeiotic germ cells [184]	Atrx variant associated with ANM [58]
	Nucleotide excision repair (*Csb* and *Xpa*) and *Fan1* are dispensible in mouse PGCs [48].	*CSB* associated with POI [177]
Fetal oocytes	Primary (fetal) oocytes deficient in synaptonemal complex and meiotic recombination genes have chromosomal defects leading to infertility. Reviewed in [81].	Refer to Table 3
	Line-1 activity triggers fetal/postnatal oocyte attrition through DNA damage-driven apoptosis [185].	
	Primordial follicles express *p53* and *p63* and respond to DNA damage by apoptosis via *Atm*-*Chek2*-*p53/p63* pathway [186], [187], [188]	
Adult oocytes	GV oocytes have a G2/M checkpoint regulated by PKA-WEE1/Cdc25B-Cdk1, which facilitates GV arrest upon DNA damage [189]	
	GV oocytes use the Spindle Assembly Checkpoint in response to DNA damage [190], [191].	
Gonocytes	Gonocytes express *Bcl-x*, *TP63* and respond to DNA damage by apoptosis [192].	Refer toTable 3
Adult spermatocytes	Spermatocytes deficient in Fanconi anemia, HR, MMR and synaptonemal complex genes show synaptic defects, pachytene arrest and DNA fragmentation leading to infertility. Reviewed in [81], [193].	Also seeTable 5
	Post-pachytene spermatocytes used the NHEJ pathway (Ku70/80) and *Ku70* mutation reduces testis size and affects spermatogenesis [194]	
	Rounds spermatids use Parp1-XRCC1 pathway to repair DNA damage [195].	
Preimplantation-embryo	*Chek1* mediated zygotic checkpoint to monitor DNA lesions on paternal genome before entering mitosis [196]	
	*MMEJ* and *NHEJ* used predominantly to repair Cas9-mediated DSB [24], [25], [26]	
	The Spindle Assembly Checkpoint is active in murine preimplantation embryos [197].	

Abbreviations: PGC-primordial germ cell; ICL-intrastand crosslink; BER-base excision repair; HR-homologous recombination; GV-germinal vesicle; MMR-mismatch repair; NHEJ-non-homologous end joining; MMEJ-microhomology mediated end joining; XPF-xeroderma pigmentosa; POI-premature ovarian insufficiency; ANM-menopause.

a Mouse studies from which inference to human conditions are made.

b Studies in human patients.

### 3.2 Fanconi Anemia-dependent interstrand-crosslink repair is critical in primordial germ cells

Germ cell hypoplasia and primordial germ cell depletion are also seen in a range of Fanconi Anemia (FA) mouse mutants [45], [46], [47], [48]. The Fanconi Anemia pathway recognizes DNA structures associated with stalled replication forks in response to interstrand crosslinks that impede DNA replication and transcription (Fig. 2-Fanconi Anemia). Besides its role in removal of interstrand crosslinks, the Fanconi Anemia proteins are also activated in response to R-loop formation (RNA: DNA hybrids) [49]. It consists of 19 genes that include homologous recombination genes such as *BRCA1/FANCS* and *BRCA2/FANCD1* (Fig. 2). Pathogenic mutations in the core complex components *FANCA, FANCC* and *FANCG* account for 85% of cases and are associated with hematological defects, subfertility, malignancies and increased spontaneous chromosome breaks and sensitivity to DNA crosslinking agents such as diepoxybutane and mitomycin C [50]. Mouse mutants deficient in *Fancm*, which recognizes DNA substrates on stalled replication forks, display increased DNA damage foci (γH2AX) and reduced number of primordial germ cells. The loss of primordial germ cells can be partially rescued by inhibiting the ATM-p53-p21 signaling cascade in males but not females [46]. Further evidence suggests that *Fanca* together with *Xpf1-Ercc1* and *Slx4/Fancp*, which are required for the repair of DNA lesions and interstrand crosslinks regulate primordial germ cell proliferation, but not differentiation or migration to the genital ridges. However, transcription coupled excision repair gene *Csb* and nuclease *Fan1* (required for repair of ICL-induced DNA breaks for efficient homologous recombination) are dispensable for primordial germ cell proliferation (Table 4) [48]. In primordial germ cells, endogenous DNA crosslinks that are substrates for Fanconi Anemia-mediated repair may derive from reactive aldehydes, since *Fanca* becomes critical in *Aldh2* and *Adh5* mutants that accumulate endogenous reactive aldehydes [51], [52]. The level of activity of the Fanconi Anemia pathway appears to be sufficient to sustain primordial germ cell numbers, at least in inbred mice, since single mutants of *Aldh2* and *Adh5* have wild-type levels of primordial germ cells by embryonic day 12.5 [48].

In humans, mutations that cause Fanconi Anemia are rare (one in 160,000 to 350,000 live births). However, patients usually present at a young age and most boys and half of the girls have hypogonadism and other urogenital defects [50], [53], [54]. In otherwise healthy males, who have non-obstructive azoospermia, biallelic mutations in *FANCM* cause a Sertoli-cell only syndrome, where the tubules are depleted of spermatogonia and spermatocytes [55]. This clinical phenotype is consistent with mouse models and could be explained by a smaller pool of primordial germ cells as well as a lower proliferation rate of gonocytes and spermatogonial stem cells [46]. It seems plausible that Fanconi Anemia genes have important roles in determining the rates of proliferation and ultimately the size of mitotically-dividing germ cell pools in human males (reviewed in [56]).

In females, Fanconi Anemia mutations have a more variable impact on fertility. The clinical phenotypes include hypogonadism and premature/primary ovarian insufficiency (POI), which is characterized by depletion of the ovarian reserve and reproductive senescence before the age of 40 (Table 3) [57]. Specifically, mutations in *FANCM* as well as *FANCA*, *BRCA1/FANCS* and *BRCA2/FANCD1* are associated with premature ovarian insufficiency [58]. The more severe impact on male fertility may be explained by the proliferative nature of primordial germ cells, gonocytes and spermatogonial stem cells that continuously generate cells that enter spermatogenesis. Thus, human reproductive phenotypes in both sexes are consistent with Fanconi Anemia being active in primordial germ cells and other proliferative germ cells.

### 3.3 Contribution of mutational processes in proliferating germ cells to human genome evolution

Several genomic features have been investigated as the source of *de novo* single nucleotide variants in humans from sequencing studies of trios (mother-father-offspring) and by correlating CpG replication timing and other chromatin marks with mutations rates (reviewed in [59]). One such genomic feature is the spontaneous deamination of 5-methyl cytosine at CpG sites, which has been suggested to cause C to T transitions. The recent development of CpG maps in human germ cells, including primordial germ cells [60], [61], have facilitated both cell-type and site-specific analyses of mutations rates and provide support for evidence that the methylation status at CpGs correlate with C to T mutations [62].

Whereas C to T transitions are passed onto the next generation and into the human population, it is less clear whether structural variants and aneuploidies that may arise in proliferating germ cells can be passed onto the embryo or whether they might be eliminated by checkpoints in germ cells. There has been substantial controversy over whether chromosome missegregation in oogonia could be a source of human aneuploidies in conceptions [63], [64]. The achievement of in vitro reconstitution of gametogenesis from induced pluripotent stem cells (iPSCs) should allow for mechanistic studies of genome instability in proliferating germ cells and their impact on gametogenesis, fertility and health in offspring [65], [66], [67].

## 4 Genome instability in human oocytes shapes fertility over reproductive lifespan

Reproductive ageing in human females is characterized by a decline in the ovarian reserve as well as elevated levels of aneuploidy in eggs that mature. DNA damage and repair influence female reproductive lifespan from fetal life to menopause [58], [68]. Aneuploidies affect up to 60% of eggs as women reach their late 30s/early 40s [69]. Meiotic recombination rates in human fetal oocytes are highly variable and are protective against aneuploidy, but mutagenic, which may ultimately limit their rates [70]. Therefore, DNA damage and repair pathways influence female reproductive lifespan from fetal life to menopause.

### 4.1 DNA damage as a determinant of ovarian ageing

In human females, the ovarian reserve is established during fetal development and is gradually depleted until menopause (Fig. 1-Female). The DNA damage response and the repair capacity of oocytes have been proposed to regulate this depletion of the ovarian reserve, resulting in menopause [58]. Human oocytes are highly sensitive to DNA damaging agents, such as those used during chemotherapy and a plethora of mouse studies have been conducted to assess effects of chemotherapy on female fertility (reviewed in [71]). This has led to programs for fertility preservation in women prior to initiating the gonadotoxic treatment [72], [73].

Endogenous DNA damage, currently of unknown sources outside of meiosis, have also been proposed to cause primary ovarian insufficiency, a pathogenic condition that affects 1% of women and is characterized by anovulation for at least four months prior to the age of 40 years (Table 3). Mutations in several DNA damage response and repair genes, including *FANCM* and *BRCA1/2* as well as meiosis-specific homologous recombination genes (*MSH5*, *RAD51*), are implicated in primary ovarian insufficiency [74], [75]. However, these mutations are generally extremely rare, represent a clinical extreme, and only explain reproductive senescence in 0.05% of all females [58]. Given the broad distribution in female ages around menopause, it is likely that the DNA repair might play a much larger role in reproductive senescence in the general population. Genome-wide association studies in the UK Biobank identified common variants in a range of DNA damage response and repair genes that individually have small effects but collectively can explain at least some of the variation in the age at which women experience natural menopause (Table 3) [58], [68]. Interestingly, the GWAS signals also include genes with important functions in meiotic recombination during the earlier stages of meiosis during fetal development (*MSH5*, *DMC1*, and *SYCP2L*); therefore, it is possible that the DNA damage repair network determines both establishment of the follicle pool as well as its depletion until reproductive senescence [58].

### 4.2 Human aneuploidies follow a U curve with female age

We have long appreciated that human aneuploidies (whole chromosome gains or losses) are a major cause of congenital disorders as well as infertility and pregnancy loss (reviewed in [7]). The vast majority of chromosome imbalances are not compatible with fetal development and live birth. The incidence of aneuploidy is 0.3% in newborns, 6.9% in stillbirths, and 50% in first trimester miscarriages, thus aneuploidy affects at least 10% of all clinically-recognized pregnancies [76]. The rate of preclinical losses due to aneuploidy are currently unknown, but might be much higher as 30% of human eggs are aneuploid [69], [77]. The 30% aneuploidy rate in human eggs is an average and does not reflect the maternal age effect. Recent studies of human eggs across female reproductive lifespan revealed that aneuploidy follows a U curve that can explain the natural fertility curve in humans. Aneuploidy rates are high in eggs from teenagers (< 20 years), improve with age (20–32 years), and then deteriorate again as women reach their mid-30s. Even in the age group with the lowest aneuploidy rate, an extremely high baseline rate of 20% of all eggs is observed (Fig. 3). In young teenagers, the larger chromosomes (chr. 1–6) tend to missegregate, whereas smaller chromosomes (chr. 21, 22) are more error-prone towards the end of fertility. The segregation errors also change with age. In teenagers, whole chromosomes tend to missegregate (meiosis I nondisjunction), whereas sister chromatids, separate at meiosis I (reverse segregation) instead of homologous chromosomes or precociously segregate (premature separation of sister chromatids) in women of advanced maternal age (Fig. 3). These observations suggest that features of meiotic chromosomes change with increasing age defining the quality of the oocyte over the reproductive lifespan of the women, which acts as a molecular timer for fertility [69].

### 4.3 Meiotic recombination is a major cause of genome instability in the female germline

Meiotic recombination occurs with the introduction and repair of programmed double-strand breaks during prophase I [78] (Fig. 2-HR). Hundreds of highly conserved DNA repair genes regulate the process [79], [80] and we have extensive knowledge of their function and reproductive phenotypes in model organisms, including yeasts, plants, and mice [81]. From more than 2000 studies on meiotic recombination and DNA repair genes, we know that phenotypes can be highly sexually dimorphic; that the checkpoint responses and apoptosis differ in male and female meiosis; and that the repair of double-strand breaks induced by the highly conserved SPO11 protein can be promiscuous, resulting in chromosomal rearrangements that affect genome function.

There are significant sex-specific differences in both the rate and location of meiotic recombination events. Human female meiosis experience on average nearly twice as many events compared to males [82]. Both double strand break induction and repair can occur via exchange of genetic material resulting in crossover products or by gene conversion resulting in noncrossover products both of which vary throughout the genome [83]. Genome-wide association studies have identified more than 50 genetic variants that are associated with recombination rates and their locations [84]. Some of these variants affect both sexes whereas others influence predominantly male or female recombination.

Two critical features make human female meiosis stand out compared to not only other organisms, but human males as well. First, human female meiosis shows a tremendous variation in recombination rates between and within individuals (Table 2). In males, crossover recombination rates range from 30 to 66 in spermatocytes undergoing meiosis and 22–28 per haploid sperm. In female meiosis the range is much broader in fetal oocytes (10-fold; 7–107) [85] and adult eggs (3–4 fold; 30–120 events). Second, despite the much higher genome-wide recombination rates in female meiosis compared to males, at least 3% of fetal oocytes contain a chromosome pair that failed to recombine (crossoverless) compared to fewer than < 0.4% in spermatocytes. These crossoverless chromosomes are also a feature that preferentially affects chromosomes 21 and 22 [82], [86]. Some of these crossoverless chromosomes might be due to failure of 20% of recombination intermediates to be resolved as crossovers, which would particularly affect the smaller chromosomes where only a few sites would be initiated [87]. Typically, the DNA damage response and meiotic silencing checkpoint should eliminate meiocytes with recombination failure [88]. Data from mouse suggest that oocytes are particularly tolerant of recombination failure compared to spermatocytes, likely due to different implementation of meiotic silencing and cell death [89], [90].

High levels of meiotic recombination appears to protect against aneuploidy, especially as women age [77], [91]. This effect is due to crossover recombination together with sister chromatid cohesion establishing physical tethers between the homologs (Fig. 1). This ‘bivalent’ structure has to remain intact during the decades long dictyate (G2/M) arrest to ensure accurate segregation at the metaphase-anaphase transition in mature eggs [69], [92], [93], [94], [95], [96], [97]. Chromosomes with fewer physical tethers, such as chromosomes 21 and 22, are more vulnerable to missegregation due to lower recombination rates or their distribution [77], [86], [91], [98]. Therefore, aberrant recombination might contribute to the high incidence of aneuploidy in human conceptions [76].

There is compelling evidence that meiotic recombination affects human genome evolution beyond creating new arrangement of existing genes (haplotypes). Aberrant meiotic recombination is associated with maternally-derived aneuploidies [99], [100]. Individuals with Trisomy 21 have lower genome-wide maternal recombination rates compared to their unaffected siblings [101] and carry common genetic variants in a broad selection of meiotic recombination genes [102]. Furthermore, breakpoints of structural variants in the human population map to meiotic recombination hotspots [103]. Several chromosomal rearrangements are consistent with non-allelic homologous recombination (NAHR) [104], [105], in which the template choice for double-strand break repair is a homologous/homeologous sequences in another part of the genome (reviewed in [106]). Such recombination events would cause a range of different chromosomal rearrangements. Because chromosomal rearrangements are rare in live births, the impact of non-allelic homologous recombination in meiosis is poorly understood. However, studying both meiotic recombination and chromosome content in single sperm, egg and embryos should allow rapid advance in our understanding of their origins. Finally, meiotic recombination is mutagenic, as events are associated with *de novo* single nucleotide variants and insertions/deletions (INDELs), sometimes at a distance [84]. It is conceivable that although homologous recombination (HR) is predominantly implemented between homologous chromosomes in meiosis that this enforcement might vary according to genomic context [107]. Several studies in budding yeast, where recombination intermediates can be followed, have shown that strand invasions are promiscous and require Bloom helicase [108], [109] and the SMC5/6 complex [110], [111], [112] for their dissolution/resolution (Fig. 2). Taken together, the mutagenic and destabilizing effects of meiotic recombination might limit genome-wide recombination rates, despite their advantage in preventing aneuploidy in female meiosis.

## 5 Genome alterations in spermatocytes and mature sperm

Our knowledge of genome instability in the male germline comes from studies of *de novo* mutations in cohorts of mother-father-child (‘trios’), male infertility, and single sperm studies. Genome instability in spermatocytes is characterized by three features. First, defective recombination is associated with non-obstructive azoospermia. Second, low genome-wide recombination rates are associated with elevated aneuploidy risk, especially of the XY chromosomes. Finally, sperm also transmit a disproportionate number of *de novo* single nucleotide variants to the embryo compared to eggs [113].

### 5.1 Altered meiotic recombination is associated with aneuploidy in sperm

Altered recombination results in aneuploidy in both male and female gametes; however, the prevalence of aneuploidy is nearly an order of magnitude lower in sperm than eggs (2.5% vs 30% Table 1). There seems to be great variation in aneuploidy rates between males, especially those with fertility phenotypes. Sperm chromosome analyses using fluorescent in-situ hybridization in azoospermic males show a high incidence of sex chromosome aneuploidies that cause Klinefelter syndrome [114]. Azoospermia is also associated with an elevated prevalence of Robertsonian translocations that elevates the risk of pregnancy loss as well as meiotic defects in their affected offspring (reviewed in [115]).

More recently, whole genome sequencing and recombination analysis of more than 30,000 single sperm cells from 22 donors revealed an aneuploidy rate ranging from 2.5% to 7% amongst healthy males [116], [117], [118]. The largest analysis, which included 31,288 sperm cells from 20 males (18–38 years) showed that all donors experienced aneuploidies in a fraction of their sperm. The sex chromosomes and acrocentric chromosomes were the most frequently affected, which is consistent with fluorescent in-situ hybridization studies [119], [120]. The spectrum of chromosomes implicated in males (chromosomes 2, 15, 20 and 21) is different than in females, where chromosomes 15, 16, 21 and 22 display the highest aneuploidy rates.

Genome-wide recombination rates were also substantially different in aneuploid sperm compared to euploid sperm. Although the recombination maps were very similar in the three studies (22–28 crossovers per sperm), all studies reported a strong correlation between crossover frequency and accurate chromosome segregation in gametes. The most pronounced effect reported was a 36% decrease in genome-wide recombination rates in sperm with chromosome gains [118]. Lower genome-wide recombination rates may cause the formation of crossoverless chromosomes and there is evidence from patients with non-obstructive azoospermia that inferred non-exchange bivalents are elevated (12.4% vs 4.2% in chromosomes 9, 21, and XY) [121]. Taken together, meiotic recombination has a major impact on chromosome segregation in spermatocytes, including in the general population.

The majority of Klinefelter (47,XXY) of paternal origin results from failure of recombination in the pseudoautosomal region (PAR) leading to XY non-disjunction [122]. The pseudoautosomal region accounts for only 0.001% of the genome, yet an obligate crossover has to form in every male meiosis. Studies in mouse have revealed several layers of regulation that ensure the obligate XY crossover. This includes a *SPO11* isoform that specifically targets double-strand breaks to the pseudoautosomal region as well as complex chromosomal modifications in heterochromatic mo-2 minisatellites, which ensures the repair outcome results in a reciprocal crossover [123], [124], [125], [126].

### 5.2 Defective meiotic recombination as a cause of male infertility

Male infertility is a multifactorial and complex disorder that affects 7% men of reproductive age. Male infertility is the reason for IVF treatment in 50% of couples. Half of all male infertility cases are idiopathic and may affect not only the male but also the female partner.

In more than 30 mouse models, defects in meiotic recombination causes male sterility due to abrogated meiosis at the pachytene stages, when homologous chromosome are normally fully synapsed and the XY pair is silenced [81]. Recent evidence support the interpretation that aberrant or disrupted meiotic recombination also causes infertility in human males. Pathogenic mutations in key meiosis-specific recombination genes such as *DMC1, SYCE1, SYCP2,* and *SYCP3* are associated with non-obstructive azoospermia (Table 4, Table 5). Furthermore, pathogenic variants in other DNA repairs genes such as *FANCM, FANCA and XRCC2* also cause meiotic arrest and non-obstructive azoospermia (Table 4, [55], [127], [128]). Besides the genetic causes, various exposures and lifestyle factors have also been reported to increase sperm aneuploidy, and understanding their molecular pathologies is important to develop appropriate public health protocols to minimize exposures as well as personalized treatment of infertile men [129], [130], [131].

**Table 5:** Pathogenic mutations in meiotic recombination and DNA repair genes in infertile male patients.

Gene	Testis histology	Semen phenotype	References
*DMC1*	Meiotic arrest	Azo	[170]
*SYCE1*	Meiotic arrest	Azo	[171]
*SYCP3*	Meiotic arrest	Azo,SO	[172]
*FANCM*	Meiotic arrest	Azo, O	[55]
*USP26*	Meiotic arrest	Azo,SO	[198], [199]
*SPO11*	Maturation arrest	Azo	[200]
*FANCA*	Meiotic arrest	SCOS	[127]
*MEIOB*	Meiotic arrest	Azo	[201]
*SYCP2*	Meiotic arrest	Azo,Cryptochridism	[202]
*XRCC2*	Meiotic arrest	Azo	[128]

Abbreviations: Azo-non-obstructive azoospermia; SCOS-sertoli cells only syndrome; SO-severe oligozoospermia, O- oligozoospermia.

### 5.3 Genome instability in conceptions from the paternal germline

Aneuploidies in sperm are passed on to the embryo and can be seen in preimplantation embryos, fetal losses and live births. The major contribution to human genome evolution, however, might derive from *de novo* single nucleotide variants, which is an order of magnitude higher in sperm than eggs. Moreover, *de novo* single nucleotide variants show a strong correlation with paternal age, with an additional two mutations accumulating per paternal year (Table 2) [132]. Since paternal age is associated with an elevated incidence of psychiatric conditions in children, it has been suggested that some of the paternal-age related *de novo* single nucleotide variants may contribute towards this effect, since they occur in genes implicated in schizophrenia and autism [132].

## 6 High rate of genome instability in zygotes and cleavage-stage embryos

Our understanding of genome instability in human embryos has been propelled by clinical development of preimplantation genetic testing as well as genome editing tools to explore early development. We review here the findings, limitations, and how genome editing and the use of Cas9-mediated double-strand breaks are advancing our understanding of genome instability during preimplantation development.

### 6.1 Human embryo development is characterized by genome instability

Genome instability in human embryos is influenced by both meiotic and embryonic factors. After fertilization, both the maternal and paternal genomes undergo extensive epigenetic reprogramming and the initial 2–3 embryonic divisions in the cleavage stage embryo is driven by maternal factors, until embryonic genome activation around day 2 to day 3 [133]. Following embryonic genome activation, the blastomeres undergo compaction in which they adhere to each other to form a cluster of cells called the morula. Subsequent cell divisions lead to the formation of the blastocyst that is comprised of a blastocoel (a fluid filled cavity) and an inner cell mass (ICM) surrounded by a layer of trophectoderm cells (Fig. 4). Implantation of human embryos on the uterine wall occurs on the 7th day of embryo development [134]. The trophectoderm cells give rise to placental cytotrophoblast cells that develop into the placenta [135]. Human embryo development occurs in a self-organizing manner and has been studied until primitive streak formation, the legal limit for in vitro development [136], [137].

Genome alterations in embryos are the result of the cumulative changes from both gametes and those occurring during preimplantation development. Preimplantation genetic testing (PGT) for monogenic disorders, structural rearrangements, and aneuploidies (PGT-M, PGT-SR, PGT-A) are now commonplace (Fig. 4). Therefore, we have extensive data on the incidence of aneuploidies (Fig. 3). To date, more than 1000 studies have been published and the field has seen tremendous advances in whole genome amplification methods, comprehensive screening, and detection of genomic changes from single nucleotide variants to aneuploidies [138].

The genomics technologies are similar to those applied to human gametes, however, both technical and biological differences are substantial. Embryos are multicellular and biopsies typically consist of a single cell (a blastomere from the 8-cell cleavage stage embryo) or an unknown number of cells (3–10) from the trophectoderm (blastocyst stage embryo) (Fig. 4). Within the preimplantation genetic testing field there has been substantial discussion about methodology and artifacts. This is important in order to separate artifacts from mitotically-derived genome alterations that arise during preimplantation development. In some cases, estimated rates vary by as much as 5-fold between studies [23], [139]. Besides the different analysis methods, embryonic cells are dividing and differentiating, whilst undergoing extensive chromatin remodeling. Therefore, heterogeneity in the DNA damage response might be expected, pending gene expression and the cell cycle stage of the cells. A final confounding issue is using infertile patients as a proxy for rates in natural conceptions. Whereas gametes can be studied in the healthy, fertile partner by selecting the participant population such as egg donors or women where the male partner is infertile, embryos will be affected by defective gametes from either. This could contribute towards the high incidence of genome instability that are observed during the cleavage stages when 40% of embryos arrest.

With these caveats in mind, several important concepts are emerging. One of the major advancements in our understanding is that aneuploidies transmitted from gametes do not appear to affect preimplantation development. However, the early embryonic divisions themselves are remarkably unstable [140], [141], [142], and may in part be due to spindle instability [141], [143] during the maternal-to-zygotic transition. As a consequence, this may give rise to diverse patterns of aneuploidy as well as mosaicism throughout the embryo [140], [144].

The cleavage stage divisions driven by the maternal factors also exhibit markers of DNA damage and replication stress that may cause embryonic arrest [145]. At least 40% of embryos arrest during this stage and the underlying causes are unclear. A recent study in mouse embryos showed that chromatin remodeling and removal of H3K9me3 inactivating marks were essential for both embryonic genome activation (EGA) and genome stability. Loss of lysine-specific demethylase 4A (KDM4A) alters the global H3K9me3 landscape in mature oocytes. Indeed several genes such as *Pif1* (5’–3’ DNA helicase)*, Wee1* (G2 checkpoint kinase) *and Rpa2* (Replication Protein 2) all critical for DNA replication and cell cycle regulation were silenced in the *kdm4a* mutant, which might explain the observed DNA damage phenotype [146].

Clinically, when couples experience high rates of embryonic arrest, it is known as ‘embryonic failure’ (Table 3). Genetic analyses of couples with embryonic failure are relatively sparse, however, one study with subsequent functional assessment identified a pathogenic mutation in *REC114*, a gene involved in the initiation of meiotic recombination. The REC114 forms a complex with MEI4 and CCDC36 to induce double strand breaks [147], [148]. Loss of REC114 function leads to decrease in double strand break formation during oogenesis and spermatogenesis [149], [150]. A few studies show that sperm DNA fragmentation is a contributing factor of embryonic failure and genome instability, though its causes remain unclear [113], [151], [152]. However, a recent study concludes that sperm DNA fragmentation does not affect fertilization, blastulation, aneuploidy, or pregnancy outcomes after in-vitro fertilization and intra cytoplasmic sperm injection [153].

A consequence of genome instability might be the formation of gross chromosomal rearrangements, also referred to as ‘segmental aneuploidies’ (dnCNVs larger than 10 Mbp), which affect about 5% of blastocyst stage embryos. Recent sampling throughout blastocyst stage embryos revealed that about two out of three were mosaic (two or more karyotypically different cell lineages in a single embryo) which indicates that these chromosomal abnormalities were not contributed by the gametes, but are a result of mitotic defects during early embryonic divisions [23]. This suggests that genome instability during preimplantation development is a major source of chromosomal rearrangements.

### 6.2 Site-specific Cas9 double-strand break induction reveals that microhomology mediated end joining is active in human embryos

Recent studies using Cas9 to induce site-specific breaks in fertilized human eggs or zygotes have revealed that DNA repair is highly mutagenic. Sequencing of repair events showed that microhomology mediated end joining (MMEJ) is the predominant double strand repair pathway used in embryos. The microhomology mediated end joining pathway repairs the double stand breaks by alignment of microhomologous sequences internal to the broken ends before joining and resulting in deletions flanking the original double strand breaks, which causes high rates of mutagenic insertions and deletions (Fig. 2-MMEJ). Another critical finding is that the induction of Cas9 breaks also results in chromosome deletions or the loss of the entire chromosome altogether [24], [25], [26]. Both the preference for mutagenic repair by microhomology mediated end joining as well as the frequent loss of the entire chromosome presents substantial scientific barriers to heritable genome editing to avoid transmission of monogenic disorders.

## 7 Perspectives

Traditionally, the ‘germline’ has been seen as the conduit of genetic information and the cause of *de novo* genetic disorders. Studies in germ cells, their precursors and preimplantation embryos are revealing a much more complex landscape. Substantial genome instability appears to be specific to the cell type and coupled to developmental changes, such as epigenetic remodeling. The prevalence of certain DNA damage repair mechanisms is analogous to somatic tissues, where both the DNA lesions and subsequent repair may be specific to the development of the cell type.

The recent technological advances in single cell sequencing have provided a revolution in our knowledge of genome diversity that is being generated in our germline and preimplantation embryos. However, we still lack mechanistic studies of the relevant human germ cells and their progenitors, something that may become feasible as in vitro systems become more developed [65], [66], [67]. Such developments would allow fundamental insights into the molecular and cellular mechanisms that diversify and safeguard our genome as it is passed on from parent to future generations. They would also facilitate the development of clinical tools for personalized genomic medicine to test suspected genetic variants for functional impact on reproductive health outcomes. One of the most revealing aspects from the studies of the recent decade is that genome instability is exceptionally high and may shape fundamental aspects of human evolution such as fertility rates and reproductive senescence.
